# The usage of complementary and alternative medicine in gastrointestinal patients visiting the outpatients’ department of a large tertiary care centre-views from Pakistan

**DOI:** 10.11604/pamj.2016.24.247.9559

**Published:** 2016-07-15

**Authors:** Ghulamullah Lail, Nasir Luck, Abbas Ali Tasneem, AyeshaAslam Rai, Syed Mudasir Laeeq, Zain Majid

**Affiliations:** 1 Department of Hepato-gastroenterology, Sindh Institute of Urology and Transplantation, Karachi, Pakistan

**Keywords:** Complimentary and alternate medicine, herbal medication, gastrointestinal symptoms, OPD, Pakistan

## Abstract

**Introduction:**

The use of complementary and alternative medicine (CAM) has increased over the last few years, and an emergent data suggests that some CAM modalities may be helpful in addressing gastrointestinal (GI) conditions. Our aim was to find out the prevalence of such practices for GI condition amongst patients visiting an OPD of a large tertiary care centre of Karachi, Pakistan.

**Methods:**

Patients visiting outpatient department of Hepatogastroenterology department at SIUT, Pakistan from March 2014 to March 2015, were included in this cross sectional study. A pre designed questionnaire was used that included the demographic data, primary disease of the patient, CAM modality used, reason for the use of CAM therapy and reasons for stopping it. Frequencies of different variables were computed using SPSS version 18.

**Results:**

906 patients were interviewed, out of which 52% (471) were males. The mean age at presentation was 39.81±12.4 years. 234 (25.8%) of the participants used one of the CAM modalities; Herbal medicine being most common one, seen in 122 (52.13%) followed by spiritual 61 (26%), and homeopathy 33 (14%). The duration of therapy was limited to six months in 161(68%), whereas 7 patients (2.9%) had prolonged duration of use of more than five years. Reasons for using CAM therapy included advice by family and friends in 66 patients (28%), personal will in 42 (17.94%), no benefit from allopathic treatment in 34 (14.5%), while high cost was the reason of use in 3(5%) of the patients. The most common reason for discontinuation of CAM was no benefit, seen in 113 patients (48.30%), followed by physician's advice in 32 (17%) patients, and side effects in 19 (8%). On the other hand 44 patients (18.80%) reported benefit from the therapy while 14 (5.9%) were still continuing with CAM modality. Among the CAM users 140 (60.09%) were un-educated or had primary education while CAM nonusers had 328 (47%) were either uneducated or had primary education only correlation reveals P value 0.004.

**Conclusion:**

Significant numbers of patients used CAM therapy. A lower level of education was associated with increased usage of CAM while cost had no major impact on its usage.

## Introduction

CAM or alternative medicines are practices and therapeutics that are not included in conventional medicine therapies. It is reported that around 50% of the developed and over 80% of the underdeveloped countries use CAM [1]. Substantial rise in CAM usage has been seen in the European countries in the last two decades [2]. Some example of CAM based therapies include massage therapies, spinal manipulation, relaxation techniques, meditation, traditional Chinese medications like tai chi, qi gong and herbal medicines [3]. CAM is a field that has a great amount of public interest so modern doctors must be aware of it [4]. The use of CAM therapies does have its costs though and around $ 34 billion are spent on it annually in the USA [5]. The various forms of CAM therapies vary according to the type of illness and the nationality of the person [6]. Its usage in the field of gastroenterology is mostly seen amongst patients with functional GI disorders [4]. Functional GI disorders amount for 40% of the visits to a GI clinic, this range from GERD to constipation to IBS [7]. One study in UK showed that CAM usage for GI symptoms and IBS was 26% and 48% respectively [8]. Nilsson K et al in their study involving 28 different CAM therapies in patients with functional GI disorders showed that a minimum of at least one therapy had been tried by all 137 patients that were included in their study [9]. ZC Yang et al evaluated the use of CAM in GI and CLD patients, revealed that 39% of these patients had used CAM therapies, with Chinese herbal medicine being the most common type of CAM therapy used by them [10].

## Methods

This questionnaire based cross sectional study was conducted in the OPD of the GI department at the Sind Institute of Urology And Transplantation (SIUT), a large tertiary care centre located in the province of Sind, Pakistan. The study period was conducted in six month duration months that is from February to August 2015. A total of 906 people were interviewed as per non probability consecutive sampling from those who visited the OPD during this period. 52% of the subjects were male while the rest were female. Those excluded were either failed to consent or mentally challenged. The included subjects were not only the local residences of Karachi city but also people visiting our OPD from cities and villages of nearly all the provinces of Pakistan. The visiting patients included men, women and children of all age groups and religion. Our questionnaire was divided into two parts; the first one involving the person's demographic details, while the second one dealt with the primary disease for which CAM used, which CAM modality used, reason for use of CAM and reason for stopping CAM. Frequencies of different variables were computed. Questions were asked and the questionnaires was filled out personally by the principal investigator himself and an informed consent was obtained prior to this. Data entry and analysis was done using Statistical Program for Social Sciences Program (SPSS) version 18.0 and frequencies, cross tabulations and chi square test were obtained.

## Results

**Figure 1:**
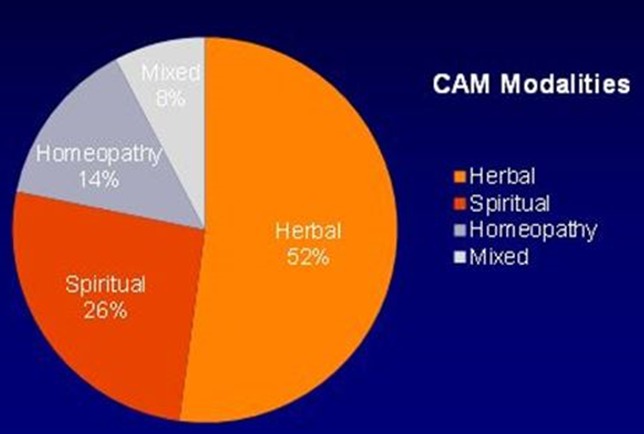
Showing the types of CAM therapies used in our patients

**Figure 2:**
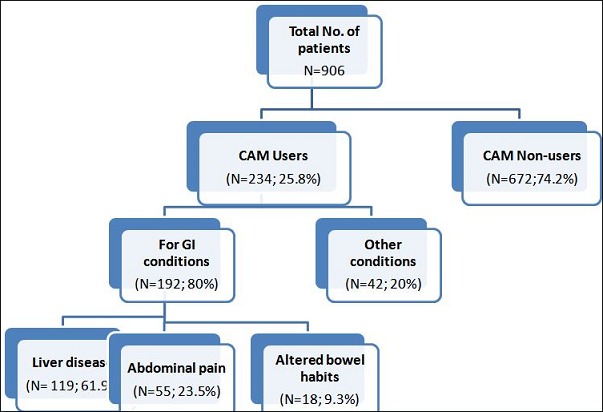
Flow chart showing indication for CAM usage as seen in our patients

A total of 906 patients were included in this study, out of which 471(52%) were males. The male to female ratio was 1.08:1. The mean age at presentation was 39.81±12.4 years. 234 (25.8%) of participants used one of the following CAM modalities(Herbal, Homeopathy or spiritual) Herbal medicine being most common one, seen in 122 patients (52.13%), spiritual therapy in 61 patients (26%), and homeopathy in 33 (14%) of the CAM users (Figure 1). The duration of therapy was limited to six months in 161(68%), whereas 7 (2.9%) reported had a duration of use of more than five years. The main reasons for use of CAM therapies included advice by family and friends seen in 66 patients (28%), personal will in 42 (17.94%), no benefit from allopathy 34 (14.5%) while high cost was the reason in 3(5%) of patients (Figure 2). The main reason for discontinuing the use of CAM was no benefit, seen in 113 (48.30%), followed by physician's advice 32 (17%) patients, and side effects in 19 (8%). On the other hand 44 (18.80%) patients reported benefit from the therapy and 14 (5.9%) were still continuing with its use. Amongst CAM users, 140 (60.09%) were un-educated or had primary education while in CAM nonusers 328 (47%) were either uneducated or had primary education. A significant P value was seen for this correlation (P value 0.004).

## Discussion

CAM's usage in the field of gastroenterology is mostly seen amongst patients with functional GI disorders [4]. Functional GI disorders amount for 40% of the visits to a GI clinic, these ranges from GERD to constipation to IBS [7]. One study in UK showed that CAM usage for GI symptoms and IBS was 26% and 48% respectively [8]. Nilsson K et al in their study involving 28 different CAM therapies in patients with functional GI disorders showed that a minimum of at least one therapy had been tried by all 137 patients that were included in their study [9]. ZC Yang et al evaluated the use of CAM in GI and CLD patients, revealed that 39% of these patients had used CAM therapies, with Chinese herbal medicine being the most common type of CAM therapy used by them [10]. Hardly any research exists on the use of CAM therapies in the pediatric GI age group. Another study showed that the use of CAM based therapies in pediatric GI patients is around 40%, with the main reason of using such therapies being non effective conventional medications, along with their adverse effects and being absent from school for more than 5 days [11].

A Hung et al in their study about the prevalence of CAM therapies in GI disease patients showed the use of CAM to be around 44% in 269 patients who were questioned, with female gender being the main group. Most common reason of using such therapies was said to be not being satisfied with the current therapy.62% of these patients even reported improvement of their symptoms after using CAM therapies and 30% of the patients did not disclosure their use to their physicians mainly because their physicians did not inquire about its use (reported by 80% of these patients) [12]. CAM based therapies are frequently used for chronic constipation. The main one used is acupuncture followed by herbal therapy [13]. In another research highlighting the use and cost of CAM in patients with functional GI disorders showed ginger to be the most common CAM used for it. CAM usage was seen more in those having a higher level of education and those having anxiety disorders [14]. The prevalence of IBS is 5-20% in the general population and the use of CAM in them leads to a better control of their symptoms [14]. More than 70% of the developing countries are in use of CAM therapies [15]. Having a rich cultural history, the use of medicinal plants for healing purpose based on the *unani* system (herbal) is very common in Pakistan and dates back to the Indus civilization [16]. While the use of CAM or alternative medicine was found to be around 70-80% in Pakistani population [17]. Studies on the use of CAM therapies in Pakistan are still in its early stages. Since most of the Pakistani population lies below the poverty line and with spiritual and religious beliefs deeply embedded in our society, the use of CAM therapies is very common in our setup [8]. One study conducted at a large tertiary care hospital in Karachi, Pakistan showed that the use of CAM therapy amongst patients visiting the health care centre was around 59%, with herbal medicine being the main type of CAM therapy followed by homeopathic [18]. Another study on the use of CAM therapy in chronic hepatitis C patients at a Rawalpindi hospital showed that the main reason of resorting to alternative medicine was the cost of conventional therapies [19]. In our study we evaluated only few modalities of CAM, the research can be expanded including *Hijmaa*, different dietary products and acupuncture.

## Conclusion

More than a quarter of patients use CAM therapies, commonest reason for use of CAM was family and friends advise while on contrary cost had no major impact on its usage. A lower level of education was associated with increased usage of CAM.

### What is known about this topic

- More than a quarter of patients use CAM therapies;
- Commonest reason for use of CAM was family and friends’ advise while on contrary cost had no major impact on its usage;
- A lower level of education was associated with increased usage of CAM.

### What this study adds

- Information on the use of CAM therapies in Pakistan;
- The most common one used;
- One of the largest studies on the use of CAM therapies in Pakistan.
